# Complete pneumothorax during subcutaneous cardiac defibrillator implantation by crocheting a rib: an uncommon complication

**DOI:** 10.1093/ehjcr/ytae483

**Published:** 2024-09-12

**Authors:** Antoine Desbiolles, Sandrine Venier, Stephane Kouame, Pascal Defaye

**Affiliations:** Department of Cardiology, Electrophysiology Unit, University Hospital of Grenoble Alpes France, CS10217, 38043 Grenoble Cedex 9, France; Department of Cardiology, Electrophysiology Unit, University Hospital of Grenoble Alpes France, CS10217, 38043 Grenoble Cedex 9, France; Department of Cardiology, Electrophysiology Unit, University Hospital of Grenoble Alpes France, CS10217, 38043 Grenoble Cedex 9, France; Department of Cardiology, Electrophysiology Unit, University Hospital of Grenoble Alpes France, CS10217, 38043 Grenoble Cedex 9, France

## Case description

A 24-year-old patient was hospitalized in our service for the implantation of a subcutaneous implantable cardioverter-defibrillator (S-ICD) in secondary prevention consecutive to an arrhythmogenic right ventricular dysplasia. The patient was rather thin with a BMI of 20 kg/m^2^.

The procedure was simple, implantation of the lead in the left pre-sternal space and subcutaneous tunnelling of the lead to the device in the submuscular left lateral thoracic compartment. At the end of the procedure, we had correct detection on all three vectors.

**Figure 1:**
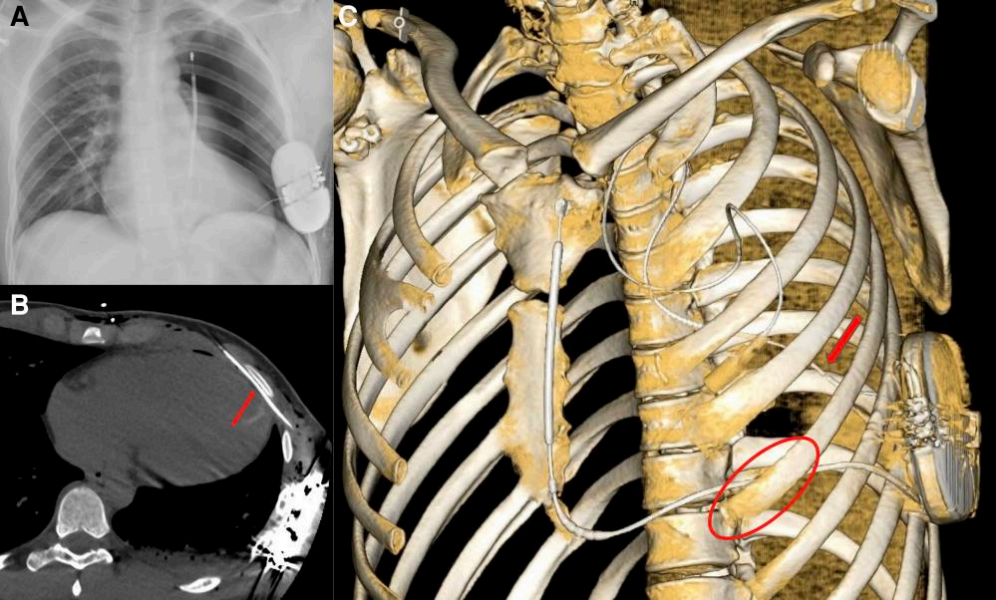
(*A*) Chest X-ray showing the complete left pneumothorax. (*B*) A computed tomography (CT) scan showing the intrapleural pathway of the lead under the 5th rib. (*C*) 3D reconstruction of the CT scan showing the subcostal path of the lead (red circle) and the thoracic drain (red arrow).

During the post-operative monitoring, the patient reported chest pain and breathing difficulties. A chest X-ray revealed a complete left pneumothorax (*Figure 1A*).

A subsequent computed tomography (CT) scan confirmed the diagnosis, finding an intrapleural pathway for the ICD lead under the fifth rib just next to the ventricular apex (*Figure 1B and C*).

The patient underwent pleural drainage, and we proceeded with the extraction and reimplantation of the involved part of the ICD lead without removing its substernal portion. Post-operative follow-up was straightforward, and the patient was finally discharged from the hospital 4 days later.

Implantation of S-ICD is recognized as a simple procedure with few adverse events,^1^ but improper lead implantation is known for causing serious complications, particularly in very slim and small patients.^2,3^

## Data Availability

The data underlying this article will be shared on reasonable request to the corresponding author.

## References

[1] Zeitler EP, Friedman DJ, Loring Z, Campbell KB, Goldstein SA, Piccini JP, et al Complications involving the subcutaneous implantable cardioverter-defibrillator: lessons learned from MAUDE. Heart Rhythm 2020;17:447–454. 31561032 10.1016/j.hrthm.2019.09.024 PMC7519585

[2] Yanagishita T, Sakamoto S, Yoshisako Y, Sasaki K, Nakatsuji T, Yoshiyama M, et al An unexpected complication of subcutaneous ICD implantation and its successful management. JACC Case Rep 2020;2:889–893. 34317375 10.1016/j.jaccas.2020.04.044 PMC8302020

[3] Petty SA, Goel R. Abdominal emergency after subcutaneous ICD implantation. JACC Case Rep 2022;4:847–850. 35912330 10.1016/j.jaccas.2022.05.006 PMC9334135

